# Study on the mechanical behavior and fracturing mechanism of rock containing two unparallel prefabricated fissures under uniaxial loading

**DOI:** 10.1371/journal.pone.0347408

**Published:** 2026-04-17

**Authors:** Jian Li, Yunyun Wei, Tie Wang

**Affiliations:** 1 Hubei Hydrogeology and Engineering Geology Investigation Institute Co., LTD., Wuhan, Hubei, China; 2 School of Physics and Electronic Engineering, Hubei University of Arts and Science, Xiangyang, Hubei, China; 3 School of Civil Engineering and Architecture, Hubei University of Arts and Science, Xiangyang, Hubei, China; 4 Hubei Key Laboratory of Vehicle-Infrastructure Collaboration and Traffic Control (Hubei University of Arts and Science), Xiangyang, Hubei, China; University of Vigo, SPAIN

## Abstract

As a natural material, rock is widely used in construction and building engineering, such as wall brick and road foundations. However, natural rock usually contains fissures and fractures, which greatly affect the long-term structural stability. In this paper, the mechanical behavior and fracturing mechanism of red sandstone containing two unparallel prefabricated fissures under uniaxial loading are studied using the finite-discrete element method (FDEM), and the modeling results are compared with the experimental results. Firstly, the uniaxial compression tests on the intact red sandstone are conducted to calibrate the input parameters in FDEM, which are validated against experimental stress-strain curves and failure modes. Then, a series of uniaxial compression numerical models of rock samples containing two fissures with different angles (*β*_2_) of the second fissure are established. Finally, the influence of *β*_2_ on the stress-strain curve, crack number and acoustic emission (AE) characteristics, failure mode, stress and displacement fields and energy evolution during uniaxial compression is discussed. Results indicate that the uniaxial compressive strength (UCS), peak strain and Young’s modulus all increase first and then decrease with the increase of *β*_2_. Especially, these indexes have a maximum value when *β*_2_ = 90°, and have the smallest value with a *β*_2_ of 180°. The material exhibits progressive failure characteristics when *β*_2_ = 180° based on AE singles but brittle failure is dominant in other cases. Generally, shear failure is concentrated at the fissure tips, while tensile failure mainly occurs in the complete zone inside the specimen. Besides, the evolution of peak strain energy with *β*_2_ is the same as the changing trend of the above indexes, but the kinetic energy shows a decreasing trend as *β*_2_ increases. The stress and displacement fields before cracking can well explain the crack propagation and intersection mechanism. The simulated failure mode and crack morphology reproduce most of the phenomena observed in the laboratory test. The results in this paper provide theoretical support for the design of deformation and bearing capacity of fractured red sandstone-based projects such as fractured rock mass tunnel.

## Introduction

**Fig 1:**
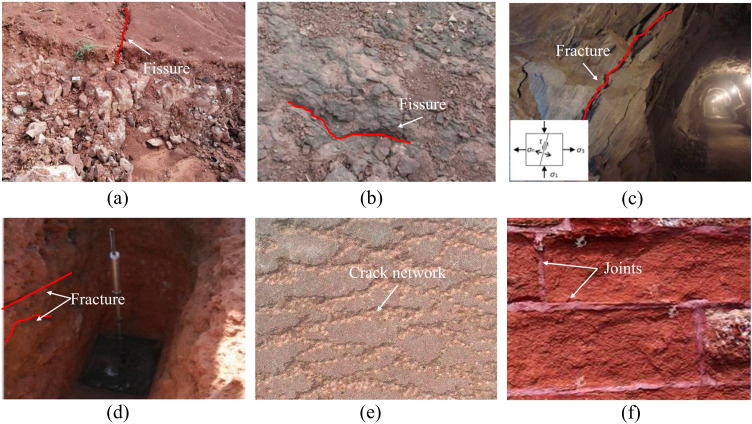
Fractured sandstone in natural and construction engineering (a)-(b) fissured red sandstone ground, (c) fractured sandstone tunnel, (d) fractured red sandstone test pit, (e) fissured red sandstone for building construction and (f) artificial jointed red sandstone wall.

Fractured rock mass is extensively distributed in various projects closely related to human life and social production, such as construction, geotechnical and energy engineering, *etc*. Compared with the intact rock mass, the mechanical properties of fractured rock mass are greatly affected by the existing fissure characteristics [1,2]. Red sandstone is a kind of natural rock building material, which is often used as building foundation, wall brick and environmental protection material and has a very wide range of applications [3,4]. Fig 1 presents the fractured red sandstone in natural and construction engineering, such as fractured red sandstone ground (Fig 1 (a), (b)), fractured sandstone tunnel (Fig 1 (c)), fractured red sandstone test pit (Fig 1 (d)), fissured red sandstone for building construction (Fig 1 (e)) and artificial jointed red sandstone wall (Fig 1 (f)). These defects have an important influence on the instability and failure of buildings and rock engineering. Meanwhile, more than one fissure exists in the rock mass with an intricate fissure network due to the complex geological and environmental effects. These fissures can lead to various accidents, resulting in huge economic and property losses, and even affecting the safety of people’s lives and property [5,6]. Therefore, studying the influence of prefabricated fissures on the mechanical properties and fracture characteristics of red sandstone is related to the safe construction of many projects, which has a practical significance for fractured red sandstone-based building engineering.

Currently, there are two main methods for investigating the mechanical properties of fractured rock: laboratory tests and numerical modeling.

In laboratory tests, the uniaxial compression test is a widely used method to study fractured rock, which can directly obtain the mechanical parameters such as peak stress, peak strain and elastic modulus of the specimen [7,8]. For example, Yang et al. [9] studied the influence of fissure orientation on the failure process and acoustic emission (AE) characteristics of red sandstone with non-parallel double fissures under uniaxial compression. The results showed that the sample has the highest uniaxial compressive strength, elastic modulus and peak strain when the fissure orientation is consistent with the loading direction. Niu et al. [10] captured the cracking process of red sandstone containing single and parallel fissures during uniaxial compression using AE and digital image correlation (DIC), and evaluated the fracture mode according to the macroscopic crack morphology. Although the DIC technique can obtain the displacement and strain field of the sample during deformation, the traditional DIC cannot measure the strain at the discontinuity. For this purpose, Miao et al. [11] developed a new DIC-based approach and successfully applied it to the displacement and strain field monitoring of red sandstone samples with single fissure under uniaxial compression. Jiang et al. [12] conducted two uniaxial compression tests on one fissure-hole rock with fissure angles of 30° and 45°, and the results indicated that the AE number of the sample with fissure angle of 45° is much higher than that with fissure angle of 30°. Besides, Wang et al. [13] performed a series of uniaxial compression tests to investigate the effect of loading rate on the mechanical properties of red sandstone with fissures and discussed the influence of fissure angle on the deformation, strength and failure mode under different loading rates. Chen et al. [14] explored the mechanical damage evolution and cracking mechanism of sandstone with prefabricated parallel double fissures under high‑humidity condition. Results indicated that the high humidity leads to the loose structure of sandstone, which creates fuzzy interfaces between layers and increases the number of micro-cracks and micro-pores. The failure mode moved from shear to tensile damage as humidity increases. Moreover, Chen et al. [15] also conducted a series of experiments on the mechanical behavior and microstructure of coal-sandstone combinations under uniaxial compression in acid dry-wet cycle conditions. It was found that the sandstone suffered more serious damage, and its pore structure and mineral composition changed more significantly, resulting in a greater decline in its mechanical properties than the coal. Sandstone is more prone to shear and spalling failure, while coal is more prone to tensile failure. The results provide new insights into the damage and degradation mechanisms of such combinations under acidic dry–wet cycles.

The above experimental work has made great efforts to reveal the mechanical behavior and fracturing mechanism of fractured red sandstone under uniaxial compression. However, there are many limitations although the experimental method is more intuitive to study the mechanical properties and failure mode of fractured red sandstone. Firstly, fractured rock is a very complex aggregate, and its failure mode is influenced by various factors. Secondly, the failure characteristics inside the rock are difficult to observe although the experimental study can directly reflect the failure characteristics of fractured rock [16,17]. Thirdly, the experimental results are often not repeatable due to expensive test funds, instruments and long cycles, resulting in the difficulty of studying the fracturing mechanism of fractured rock [18–20].

With the rapid development of computer technology, numerical modeling has gradually been employed in the field of rock and geotechnical engineering. Many scholars have applied the numerical approach to study the mechanical properties of fractured rock mass [21–23]. Reasonable numerical work also has guiding significance for practical engineering construction, which can make up for the deficiencies in experimental research [24]. At present, the main numerical methods used to study the mechanical properties of fractured rock include continuum theory and dis-continuum theory.

In the continuum theory-based approaches, Li and Wong [25] combined the finite element method (FEM) and the nonlinear dynamic method to simulate the uniaxial compression of single- and double-fractured specimens, the influence of crack angle and loading rate on the mechanical properties and fracturing mechanism were discussed. The simulation results indicated that the fissure angle and length greatly affect the failure mode. Tensile cracks were generated earlier with a small loading rate, while shear cracks appeared earlier with a large loading rate. Xie et al. [26] used the extended finite element method (XFEM) to study the effect of surface friction on crack initiation and propagation and found that the friction force of the crack surface has no obvious effect on the crack generation, while the friction angle significantly influences the crack length. Based on the XFEM and AE technology, Wang et al. [27] discussed the influence of concentrated load and uniform load on the mechanical behavior and failure mode of red sandstone samples with prefabricated fissures under bending and validated the modeling results by comparing with the experimental results.

In the dis-continuum theory-based approaches, Jiang et al. [12] carried out uniaxial compression tests on one fissure-hole rock with fissure angles based on particle flow code (PFC2D) simulations and indicated that the elastic modulus and peak stress increase gradually as the fissure angle increases from 0° to 90°. Huang et al. [28] also adopted PFC to study the mechanical behavior and AE characteristics of red sandstone with two parallel fissures under uniaxial compression. Yang et al. [29] investigated the failure mode and fracturing mechanism of double-fractured red sandstone under uniaxial compression using the discrete element method (DEM), and analyzed the influence of the second fissure angle on the mechanical properties of the material. To study the influence of the strike-dip of structural surfaces on the deformation and fracture of fractured rock mass, Kulatilake et al. [30] proposed a three-dimensional linear elastic orthotropic constitutive model in three-dimensional distinct element code (3DEC) to explore the deformation properties of jointed rock mass before failure.

Given the above literature review, continuum theory-based approaches such as FEM are difficult to accurately capture the initiation and propagation of cracks due to the fixed mesh when simulating solid cracking. It usually requires mesh rezoning or the use of specific techniques (such as XFEM), which increases computational complexity and cost. In addition, FEM may have problems of numerical oscillation or accuracy degradation when dealing with fracture problems. Although dis-continuum theory-based approaches such as DEM are good at dealing with large deformation and granular materials, it has a large amount of calculation when simulating the initiation and development of micro-cracks in continuous media. DEM parameter calibration may also be difficult when dealing with complex contact and block interaction. Consequently, there are still some limitations in the above numerical analysis for the mechanical properties of fractured red sandstone.

The finite-discrete element method (FDEM) has the common function of solving solid deformation and stress by FEM and treating contact and cracking by DEM. This approach has unique advantages in simulating rock from continuous deformation to discontinuous deformation and cracking under external loading and is more and more widely adopted to solve rock engineering problems. In 1995, Munjiza et al. [31] proposed FDEM and introduced the principles of FDEM comprehensively. Subsequently, many scholars have made great efforts to improve the application scope and function of this method. For example, Rougier et al. [32] verified the correctness of 3D-FDEM in simulating rock cracking and stress waves by laboratory tests. Lei et al. [33] developed the large-scale parallel framework theory of 2D-FDEM, which enables FDEM to solve large-scale engineering problems. Tatone and Grasselli [34] proposed an efficient method for parameter calibration of 2D-FDEM by comparing the mechanical behavior of simulated rock under uniaxial compression and Brazilian splitting with the experimental results. Li et al. [35] investigated the crack initiation and propagation mechanism of brittle rock under triaxial compression using FDEM and discussed the influence of size effect on the simulation results. Sun et al. [36] introduced the implementation of the thermo-mechanical (TM) model in FDEM for modeling fracturing of low-temperature frozen rock mass. Wu et al. [37] proposed a THM model based on 2D-FDEM and validated the proposed coupling model through several analytical examples. Yan et al. constructed numerous coupled models in the FDEM-based software of MultiFracS [38–41], which expanded the application range of FDEM in simulating cracking by multi-physics factors. Compared with the FEM-based multi-field theoretical framework [42], the FDEM multi-field coupled model can not only simulate the deformation of rock and soil, but also simulate the cracking problem driven by multi-physical fields. For example, Wang et al. studied the large deformation and cracking mechanism of soft rock tunnels [43], water absorption-induced rock microcracking [44], the effect of water on rock mechanical properties [45], rock breaking by TBM indentation [46,47], linear cutting [48] and mechanical properties of bedded rocks [49], respectively. Moreover, some other scholars also have made great efforts to the development of FDEM and its application in the rock [50–53], geology [54–56] and energy [57–59] engineering fields. However, the mechanical behavior of fractured red sandstone is not studied in the above FDEM modeling works. Thus, we aim to investigate the mechanical behavior and fracturing mechanism of red sandstone with two unparallel prefabricated fissures under uniaxial compression with FDEM. The results are expected to provide theoretical support for the construction design of deformation and bearing capacity of fractured red sandstone-based projects such as fractured rock mass tunnel. All simulations are performed in MultiFracS.

This paper is organized as follows. Section 2 briefly introduces the FDEM principles. In Section 3, the uniaxial compression numerical test of the complete red sandstone is performed to calibrate the parameters input in the model, and the stress-strain curve and failure mode are compared with the indoor results. Meanwhile, a set of red sandstone models with different fissure orientations are established based on relevant experiments. Section 4 presents the results and discussion, which focuses on the analysis of the stress-strain curve and mechanical properties of red sandstone during the loading process, crack number, AE characteristics, stress and displacement fields before cracking, failure mode, and energy evolution. The modeling results are compared with the experimental results. The main conclusions of this paper are summarized in Section 5.

## Model and methodology

### Overview of FDEM

**Fig 2:**
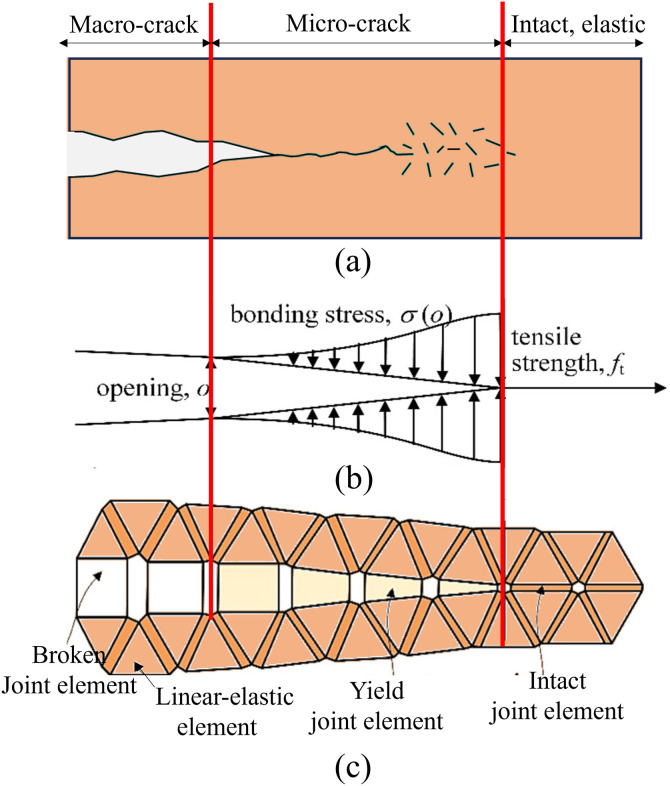
Principle of FDEM (a) continuum failure process, (b) failure process zone (FPZ) model, (c) characterization of crack propagation in FDEM (modified after Munjiza et al. [31]).

The finite-discrete element method (FDEM) was originally developed by Munjiza et al. [31]. Fig 2 illustrates the principle of FDEM. The failure of the material is usually characterized by the propagation and intersection of cracks, as shown in Fig 2 (a). Based on the failure process zone (FPZ) model (Fig 2 (b)), the continuous material is composed of triangular elements in FDEM, which are connected by an initial thickness-free virtual joint element, as shown in Fig 2 (c). The failure process of the continuous material is transformed into the fracture of the joint elements between the solid elements to simulate the crack initiation and propagation process. Since red sandstone is very sensitive to environmental changes, micro cracks and micro pores are generated in red sandstone under high humidity condition, leading to a loose structure [60]. The FPZ model in FDEM can also consider the humidity effect. The distribution of the humidity in the whole continuum can be expressed by the humidity of each triangular element node. The humidity in the triangular element can be obtained by linear interpolation. A detailed introduction of the coupling model can be found in our published studies [40,43,44].

### Fracture constitutive models of joint element

**Fig 3:**
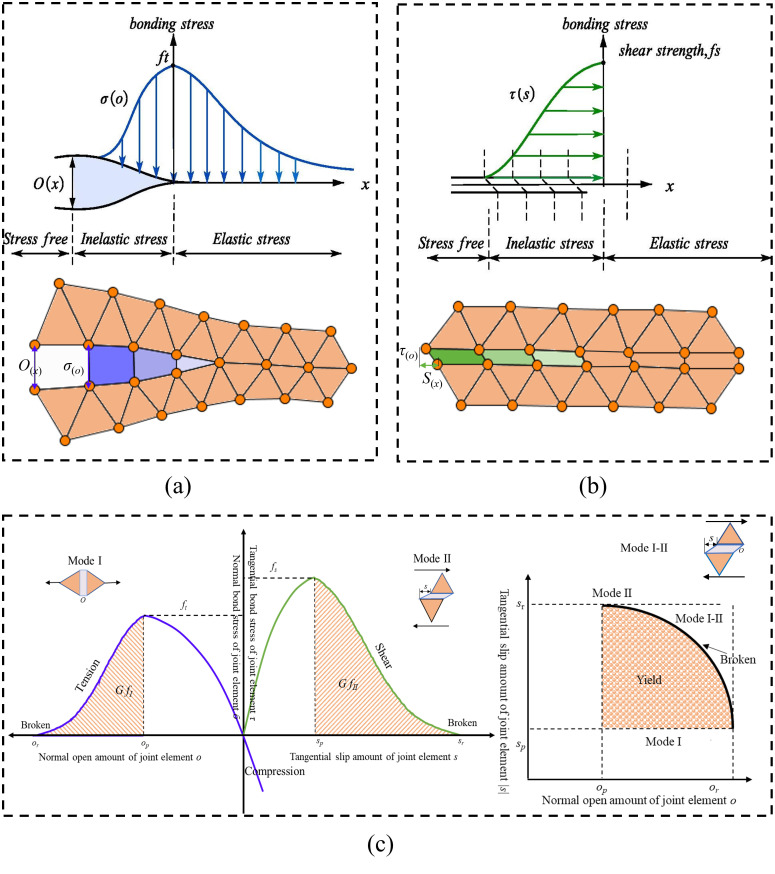
Fracture constitutive model of joint element. (a) conceptual model of FPZ for tensile damage and (b) shear damage, (c) FDEM fracture model. (modified after references [31,34]).

As introduced in Section 2.1, the joint element has the function of bonding solid elements. There are three fracture constitutive models of joint element, as illustrated in Fig 3.

(1) Model I: The normal bonding stress *σ* reaches the tensile strength *f*_*t*_ once the normal opening *o* of the two elements’ edges reaches a critical value *o*_*p*_, as shown in Fig 3 (a). Then, *σ* drops as *o* increases. Until *o* reaches the maximum opening *o*_*r*_, *σ* reduces to 0, which means that the joint element is damaged.
(2) Model II for shear failure: The shear damage has a similar evolution of bonding stress with displacement to that of tensile damage, as shown in Fig 3 (b).
(3) Besides, both open and sliding may appear for the joint element instead of the above two main fracture models, as presented in Fig 3 (c). If *o* and *s* have the following relationship in Eq. (1), mixed tensile-shear failure is generated, which is also called Mixed model I-II for tensile-shear failure:
  $${\left(\frac{o-o_{p}}{o_{r}-o_{p}}\right)}^{\text{2}}+{\left(\frac{s-s_{p}}{s_{r}-s_{p}}\right)}^{\text{2}}\geq 1$$ (1)

## Parameter calibration and model setup

### Parameter calibration

Parameter calibration is one of the most important steps in numerical simulation, which determines the correctness of the simulation results. Therefore, the input parameters in the model must be calibrated before the subsequent numerical work. As Wang et al. [46] and Zhao et al. [61] reported, the physical parameters of real rock, such as Young’s modulus and tensile strength, obtained in the laboratory test can be used as the mechanical parameters input in FDEM if the normal and tangential penalties are set to 100 times the elastic modulus. Thus, we use the mechanical parameters of red sandstone provided by Yang et al [4], who performed the basic laboratory rock mechanical tests. These mechanical parameters are: *E* = 10.6 GPa, *f*_*c*_= 54 MPa, *v* = 0.25 and *μ* = 30°. In this way, only two fracture energies of the joint element need to be calibrated, which are Type I-fracture energy (*Gf*_I_) and Type II-fracture energy (*Gf*_II_).

**Fig 4:**
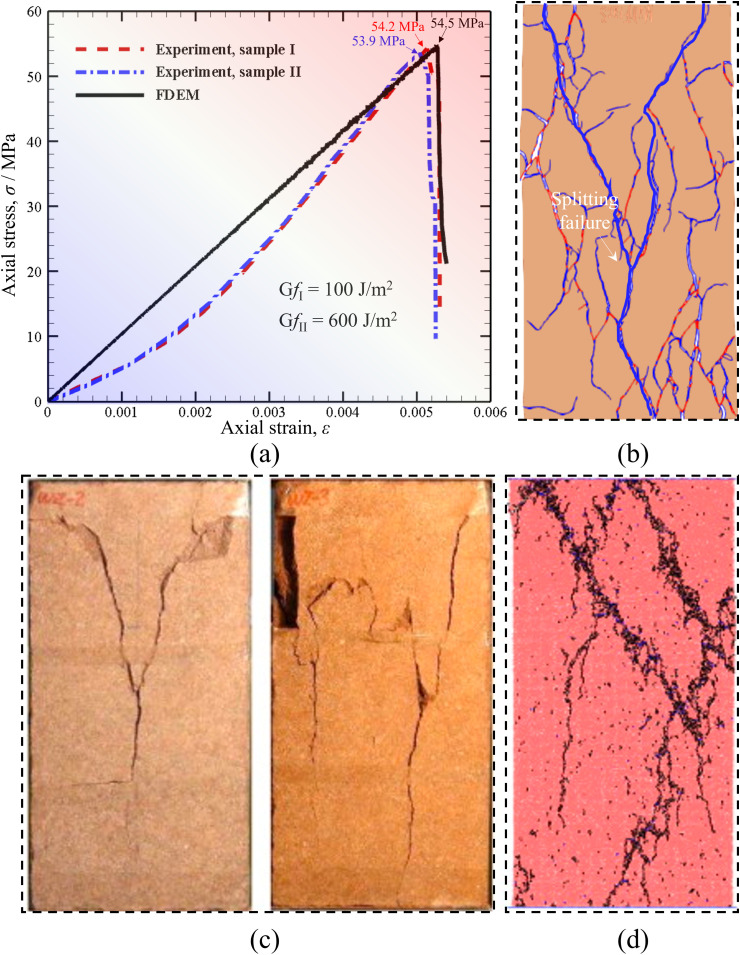
Parameter calibration via the uniaxial compression numerical test on complete red sandstone. (a) comparison of the stress-strain curves obtained by FDEM and experiment [9], (b) FDEM simulated failure model (blue-tensile, red-shear), (c) experimental failure modes [9], (d) DEM simulated failure mode [29].

As shown in Fig 4, *Gf*_I_ and *Gf*_II_ are determined here via the uniaxial compression numerical tests on complete red sandstone. Fig 4 (a) illustrates the comparison of the stress-strain curves obtained by FDEM and experiment. We find that the uniaxial compression stress-strain curve of red sandstone sample obtained by FDEM matches well with the experimental results with fracture energy values of 100 J/m^2^ and 600 J/m^2^. The uniaxial compressive strength obtained by FDEM in Fig 4 (a) is 54.5 MPa, while that obtained in the experiment are 54.2 and 53.9 MPa, and the error is only 0.55% and 1.11%, respectively. There is a little shift between the simulated and experimental stress-strain curves because the used FDEM incorporating current fracture constitutive models of joint element cannot model the pre-compression process of rock under uniaxial compression. Meanwhile, the failure mode in Fig 4 (b) indicates that splitting failure is dominant in the red sandstone under uniaxial compression, which is also consistent with the experimental results (Fig 4 (c)). Besides, Yang et al. [29] also simulate the failure mode of red sandstone under uniaxial compression based on DEM, as shown in Fig 4 (d). Obviously, the failure mode of red sandstone obtained by FDEM in this paper is more consistent with the experimental results compared with the DEM results, which also proves the correctness of the parameter calibration and the superiority of the FDEM method.

Given the above analysis and description, the fracture energies calibrated by the uniaxial tensile numerical tests of sandstone samples are *Gf*_I_ = 100 J/m^2^ and *Gf*_II_ = 600 J/m^2^, respectively. These parameters will be used in subsequent numerical calculations to discuss the influence of the prefabricated fracture angle on the mechanical behavior and failure mode of red sandstone under uniaxial compression.

### Model setup

**Fig 5:**
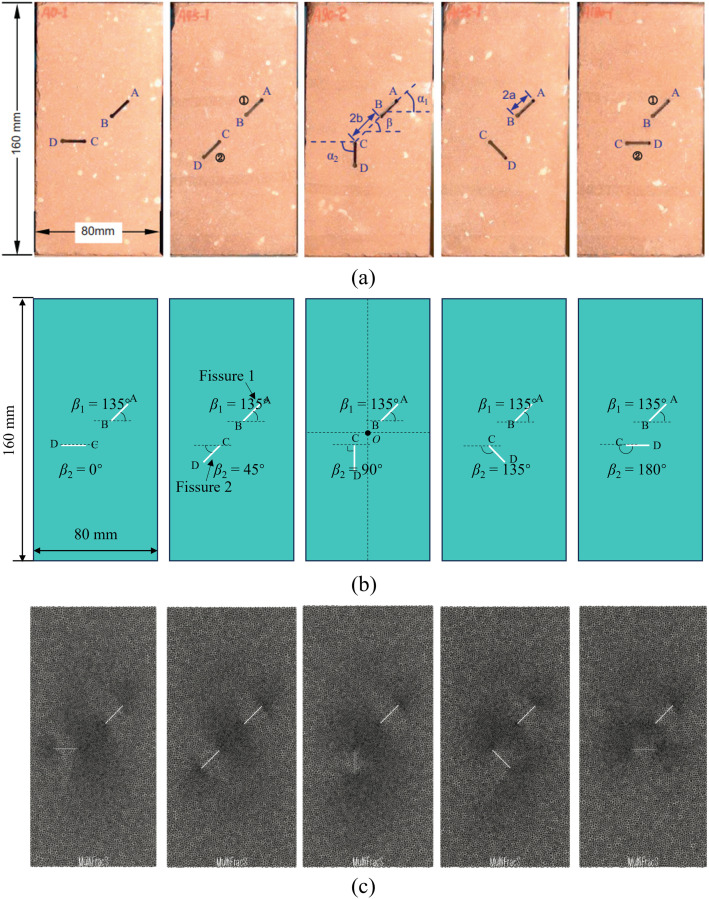
Numerical model. (a) Red sandstone pattern with different fissure orientations used in the test [9], (b) model setup, and (c) computational mesh in FDEM.

In this section, the setting of the numerical model is completely consistent with the sample size and fissure characteristics used in the test of Yang et al. [9]. As shown in Fig 5 (a), the experimental rectangular red sandstone sample has a width of 80 mm, a height of 160 mm and a thickness of 30 mm. High-pressure water jet was adopted to prefabricate two open fissures AB and CD, which are in the middle of the sandstone sample and have a length of 16 mm and a width of 2 mm. To facilitate the subsequent analysis, fissures AB and CD are referred to as fissures 1 and 2. The distance between the two crack tips, *i.e.,* the BC length is 22 mm. The orientation of fissure 1 (*β*_1_) is kept unchanged at 135°, while the orientation of fissure 2 (*β*_2_) is set to 0°, 45°, 90°, 135°, and 180° respectively to study the mechanical behavior and failure mode of red sandstone with two unparallel prefabricated fissures under uniaxial compression.

A series of numerical models with different orientations of fissure 2 are built in Fig 5 (b), where the size of the model and the geometric characteristics of the prefabricated fissures are consistent with the experiment. The loading rate is set to 0.05 m/s. Although this value is much larger than that in the indoor test, the mechanical time step used in the simulation is 1 × 10^−8^ s/step, and the loading displacement per step is only 5 × 10^−10^ m. This value is small enough to ensure that the system is quasi-static during the comprehensive process. The computational mesh used by FDEM is presented in Fig 5 (c), where the whole model is divided into 19825 solid elements and 27050 joint elements, with an element size of 1.5 mm. It should be noted that mesh refinement is taken at the two unparallel prefabricated fissures tip to more accurately simulate the crack initiation process at the prefabricated fissures.

## Results and discussion

### Stress-strain curves and mechanical properties

**Fig 6:**
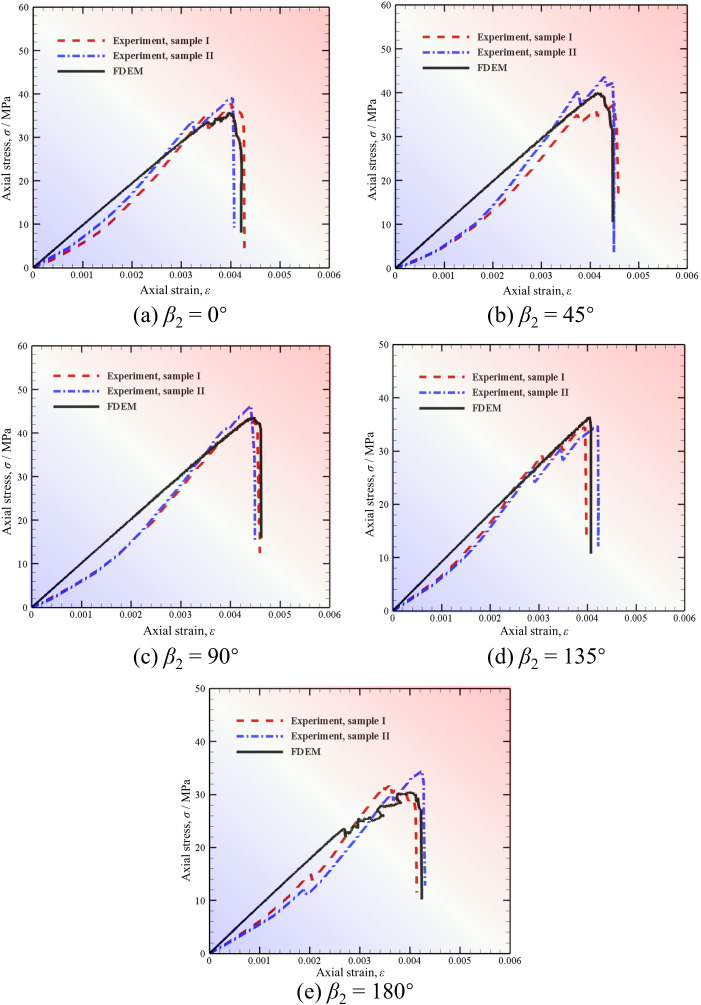
Comparison of axial stress-strain curves with different *β*_2_ between FDEM modeling and experimental results [9].

Fig 6 illustrates the comparison of axial stress-strain curves with different *β*_2_ between FDEM modeling and experimental results. It should be noted that three groups of tests were carried out for each *β*_2_ in the experiment because the sample size and meso-structure cannot be guaranteed to be completely consistent. If the error of the stress-strain curves measured by the three groups of tests was small enough, the test results could be considered reasonable [9]. Two stress-strain curves for each *β*_2_ are given here.

Fig 6 (a)-(e)– indicates that the simulation results reproduce the test results well. Specifically, the stress-strain curves of red sandstone with two unparallel prefabricated fissures with different *β*_2_ under uniaxial compression obtained by the FDEM fit well with those measured in laboratory tests. Overall, the axial stress increases with the axial strain in the early compression stage. With further loading, the stress growth rate becomes larger due to the compaction of voids and prefabricated fissures inside the red sandstone. Before reaching the peak stress (uniaxial compressive strength), the stress-strain curve becomes no longer smooth but presents a certain vibration due to the micro-cracks generated inside the sample and the extrusion and slip of rock particles. This phenomenon is particularly obvious when *β*_2_ = 0° and 180°. After reaching the peak stress, the stress begins to decrease suddenly as the further increase of strain due to the completely breaking of red sandstone.

**Fig 7:**
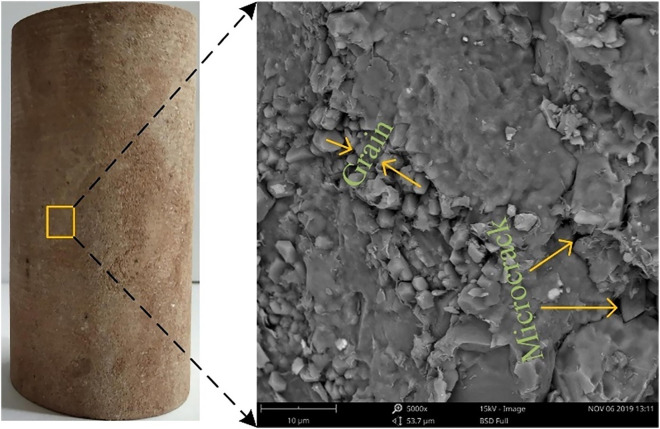
SEM image of red sandstone [61].

Furthermore, it is worth mentioning that the stress-strain curve obtained by the laboratory test is not completely linear since the real red sandstone has defects such as micro-cracks and voids based on the scanning electron microscope (SEM) image [61], as shown in Fig 7. The initial stress-strain growth rate is relatively small because these defects are gradually close during the initial compression process. Once these defects are fully compacted, the red sandstone can be considered as an elastomer, and then the stress increases linearly with the strain. However, the stress-strain curves simulated in this paper are linear before reaching the peak stress, because the FDEM used here cannot consider the micro-cracks and voids in the red sandstone.

However, some scholars have improved the constitutive model of FDEM and made FDEM simulate the micro-cracks and void closure behavior of solid materials such as rock and concrete in the early stage compression, which can better reflect the true compression characteristics of materials. For instance, Wu et al. [62] developed an image model considering rock microstructure in 2D-FDEM. Cohesive elements with a certain thickness were inserted into the model to consider the width of initial microcracks, and the rock’s nonlinear mechanical characteristics during uniaxial compression and Brazilian splitting loading were captured. Ye et al. [63] characterized the micro-cracks inside the rock sample by randomly scaling the solid elements in the 3D-FDEM and there was a certain distance between the solid elements that were originally contact closely. The simulated stress-strain curves agree well with the experimental results. Zheng et al. [50] proposed a modified joint element constitutive model in 2D-FDEM, which successfully simulated the nonlinear mechanical behavior of rock under loading by introducing a randomly opened fracture joint element instead of the micro-cracks and voids existing in the rock. The above work has made great contributions to FDEM in simulating the nonlinear mechanical behavior of rocks.

**Fig 8:**
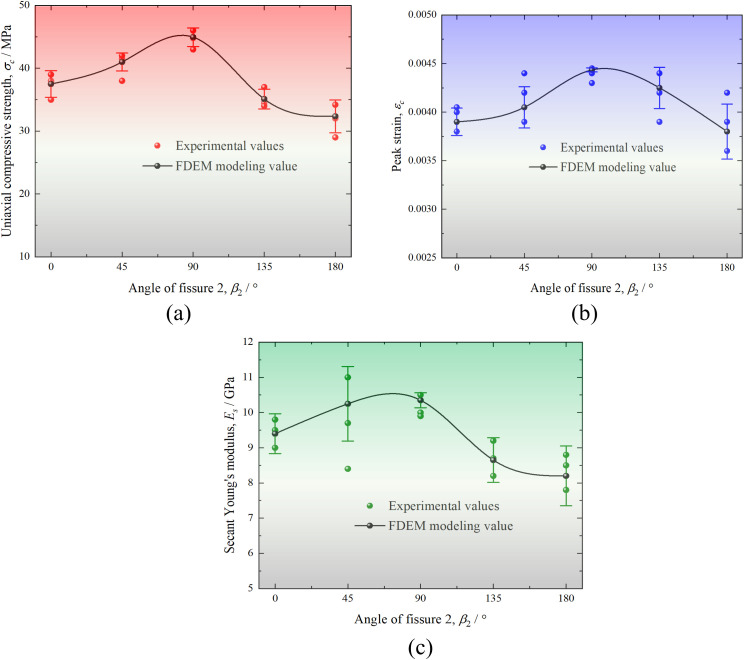
Comparison of the evolution of (a) UCS, (b) peak strain (c) secant Young’s modulus with *β*_2_ obtained by FDEM simulations and experimental tests [9].

Fig 8 compares the evolution of uniaxial compressive strength (UCS), peak strain, and secant Young’s modulus with *β*_2_ obtained by FDEM simulations and experimental tests quantitatively. Note that the secant Young’s modulus in this paper refers to the slope of the line from the peak point to the origin. As we can see, the variation trend of the above three indexes with *β*_2_ obtained by FDEM is highly matched with the experimental results. According to the error bars analysis, the relative error range of UCS in experimental values and numerical results is 4.57% ~ 8.77%, that of peak strain is 0.67% ~ 9.25%, while that of secant Young ‘s modulus is 2.86% ~ 11.25%. Therefore, the errors of UCS and peak strain is very small, both within 10%. The maximum error of the secant Young ‘s modulus reaches 11.25% because the current joint element constitutive model used in FDEM cannot simulate the pore compression behavior of sandstone at the initial stage of loading, resulting in linear stress-strain curves in the simulations.

Generally, the UCS, peak strain and secant Young’s modulus all increase first and then decrease with the increase of *β*_2_. Especially, these three indexes have the maximum values with *β*_2_ of 90°, which are about 44 MPa, 0.0044 and 10.45 GP, respectively. When *β*_2_ = 180°, the UCS and secant Young’s modulus have minimum values, which are 30.5 MPa and 7.6 GPa, respectively. The peak strain has a minimum value of 0.004 when *β*_2_ is 0° and 180°. Specifically, the UCS of the red sandstones with different *β*_2_ is 36, 40, 44, 36.5 and 30.5 MPa, respectively, which are 33.33%, 25.93%, 18.52%, 32.41% and 43.52% lower than that of the complete sample (54 MPa). On the other hand, the corresponding secant Young’s modulus are 9, 9.45, 10.45, 9.1 and 7.6 GPa, respectively with a reduction rate of 15.09%, 10.85%, 1.43%, 14.15% and 28.30% compared with that of the complete sample (10.6 GPa). Consequently, the UCS and secant Young’s modulus of the red sandstone with prefabricated fissures are the closest to that of the complete sample when *β*_2_ = 90°, indicating that the strength and deformation properties of the red sandstone with prefabricated fissures are the least affected by the presence of fissure 2 when its orientation is consistent with the loading direction.

### Crack number and AE characteristics

During the compression process of red sandstone with prefabricated fissures, new cracks will be generated when the stress reaches the material strength. These newly generated cracks will propagate and intersect, eventually leading to failure. The development of crack is often accompanied by the occurrence of AE events. Therefore, it is necessary to quantify the crack number evolution and AE characteristics of fractured red sandstones during the loading process. In this way, it can be intuitively observed when the crack is initiated, which is an advantage of numerical simulation compared with the indoor test.

**Fig 9:**
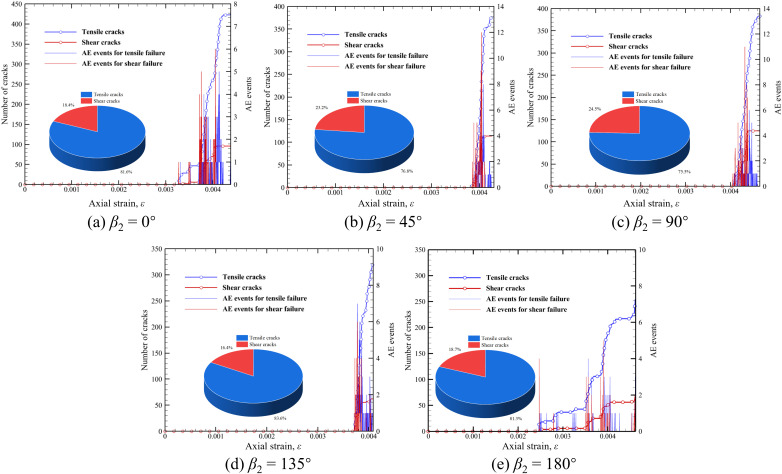
Evolution of crack number and AE signals with axial strain in red sandstone with different *β*_2_.

Fig 9 illustrates the evolution of crack number and AE signals with axial strain in red sandstone with different *β*_2_. It should be mentioned that the AE count in FDEM is defined as the number of broken joint elements in each step, which is equal to the cumulative number of broken joint elements at the current step minus the cumulative number of broken joint elements in the previous steps. Overall, no cracks and AE signals are generated in the red sandstones with two unparallel prefabricated fissures at the initial compression stage. As the sample is further compressed, both tensile and shear cracks are initiated, accompanied by many AE signals of tensile and shear failures. Combined with Fig 6, the axial strain when the acoustic emission signal is concentrated corresponds to that of the peak stress, indicating that most cracks are generated at this moment. This is consistent with the DEM simulation results [29]. Moreover, the cracks are initiated earliest with an axial strain of 0.0025 when *β*_2_= 180° (Fig 9 (e)), which indicates that the red sandstone is easiest to crack during compression, and the sample has the lowest UCS in this case, as shown in Fig 7 (a). Meanwhile, an AE signal of shear failure is generated, indicating that shear crack is generated at the prefabricated fissure tip. Furthermore, the AE signal heights for *β*_2_= 180° are relatively small, but the duration interval is larger than that in other cases, indicating that the cracks are continuously initiated and the sample presents progressive failure characteristics. However, the cracks are initiated at the latest with an axial strain of 0.0041 when *β*_2_ = 90° (Fig 9 (b)). It means that when the orientation of prefabricated fissure 2 is consistent with the loading direction, the red sandstone is most difficult to crack under uniaxial compression. In this case, the sample has the highest UCS, as illustrated in Fig 8 (a). When *β*_2_ = 45°, 90° and 135°, the entire interval of AE singles is very small, and the crack number increases rapidly in a short time (Fig 9 (b)–(d)), which corresponds to the stress-strain curve in Fig 7 (b)–(d). It should be noted that the number of tensile cracks is far more than that of shear cracks for five different *β*_2_, indicating that brittle tensile (splitting) failure is dominant in the red sandstone with two unparallel prefabricated fissures under uniaxial compression, which is consistent with the test results [9].

Furthermore, Fig 9 also indicates the final tensile-shear crack ratio distribution in five cases. We can find that the shear crack ratio is the highest *β*_2_ of 90°, reaching 24.5%. Generally, the specimen is dominant by tensile failure in five cases.

**Fig 10:**
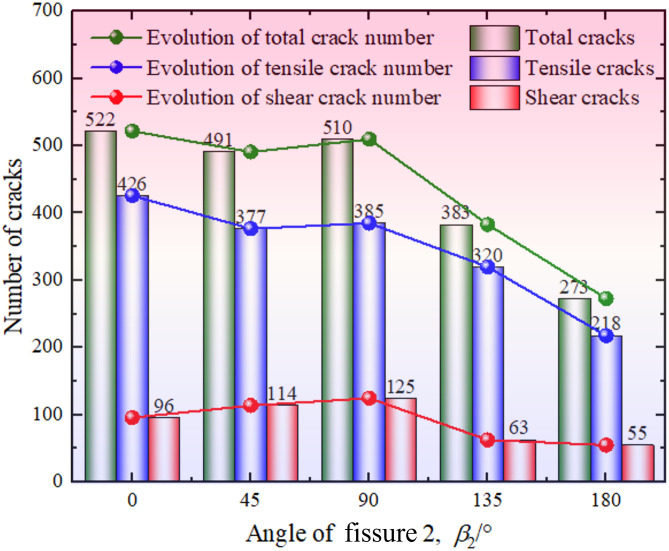
The relationship between the ultimate crack number and *β*_2_.

The relationship between the ultimate total, tensile, and shear crack number and *β*_2_ is illustrated in Fig 10, where *β*_2_ greatly affects the ultimate crack number. Specifically, the number of total and tensile cracks decreases gradually with the increase of *β*_2_ except for *β*_2_ = 90°, demonstrating that an increasing orientation of fissure 2 reduces the damage degree of the sample. However, the number of shear cracks presents a first increasing and then decreasing trend with the increase of *β*_2_, and a *β*_2_ of 90° leads to the largest number of shear cracks, which is 125. Besides, the number of tensile cracks is greatly larger than that of shear cracks, indicating that the red sandstone with two unparallel prefabricated fissures studied in this paper is mainly dominated by tensile failure under uniaxial compression. Generally, the red sandstone has the characteristics of brittle fracture if tensile failure is dominant during compression. Therefore, the red sandstone studied in this paper is a brittle material. The above analysis is consistent with the previous DEM modeling results [29].

### Stress and displacement fields before cracking

Previous works indicate that stress is first concentrated on the defects during the loading. Crack first initiates at the defects once the concentrated stress reaches the material strength [29,64]. Therefore, the analysis of the stress and displacement fields near the prefabricated fissures of red sandstones before cracking can better understand the mechanism of crack initiation and propagation. In this section, the major principal stress and major shear stress near the two unparallel prefabricated fissures before cracking are first analyzed, and the displacement field and vector are then described.

**Fig 11:**
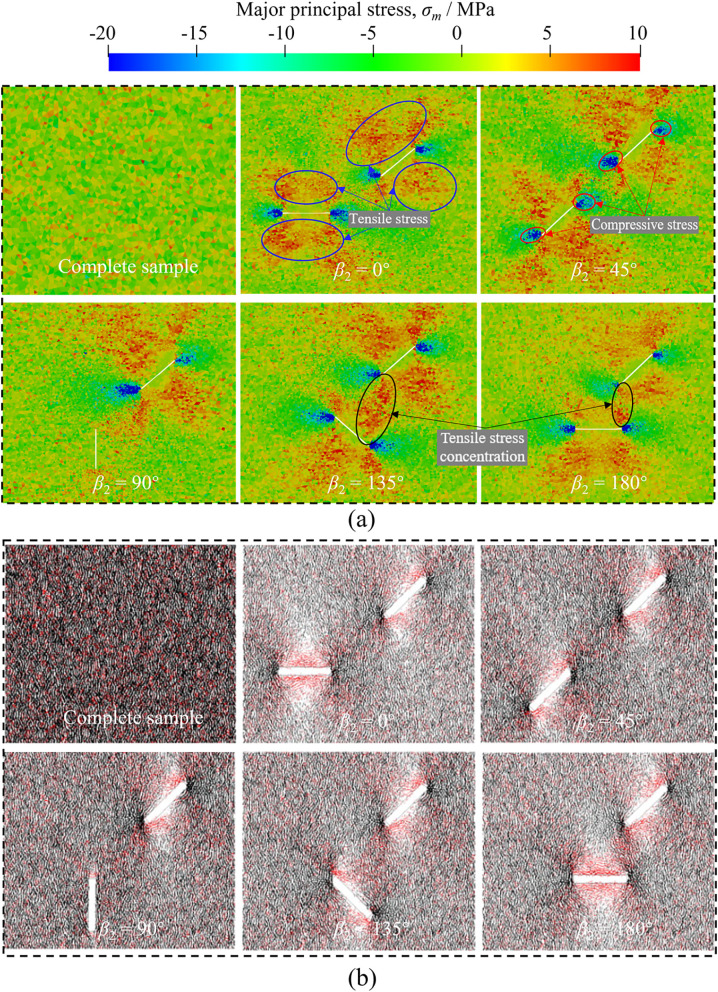
Comparison of stress distribution around fissures obtained by FDEM and DEM. (a) Distribution of major principal stress and (b) bonging forces [29] near the prefabricated fissures before cracking of red sandstones.

Fig 11 (a) presents the distribution of major principal stress near the prefabricated fissures before cracking of red sandstones with different *β*_2_. It should be noted that no crack initiates at the prefabricated fissure tips before cracking. Otherwise, the formation of the crack releases some concentrated stresses. In Fig 11 (a), a positive major principal stress represents that the sample is under tension state (red zone), while a negative major principal stress represents that the sample is under compression state (blue zone). For the intact specimen, the major principal stress before cracking is randomly distributed. However, the major principal stress is not distributed randomly before cracking in fractured specimens, and its distribution characteristics depend on the orientation of facture 2 (Fig 11 (b)–(e)). As we can see, the tips of prefabricated fissures are covered by blue, while the parts on both sides of the cracks are red when *β*_2_ = 0° and 180°, which indicates that compressive stress is concentrated at the tips of prefabricated fissures and both sides are tensioned. In addition, the tensile stress near the two fissures is much higher than that far away from the two fissures, which is consistent with the previous DEM simulation results [29,64]. When *β*_2_ = 45° and 135°, the compressive stress is concentrated at the tip C bottom and tip D top, while the tensile stress is distributed not only around the fissures but also at the tip C top and tip D bottom. However, the surrounding zone of fissure 2 is dominated by compressive stress, while the tensile stress is mainly distributed at the tip when *β*_2_ = 90°. Meanwhile, we note that both the compressive and tensile stress around fissure 2 with *β*_2_ of 90° is much smaller than that in other cases, which means that a *β*_2_ of 90° has little effect on the stress distribution of the fractured red sandstone under compression, and crack is not first initiated around the fissure 2 in this case.

Additionally, the compressive stress is mainly distributed in the straight-line zone between tips B and C with *β*_2_ of 0° and 45°, which is much smaller than that at the tips of two fissures, indicating that the shear crack is difficult to develop in the straight-line zone. When *β*_2_ = 90°, compressive stress is obviously concentrated at the tip B bottom, and the concentrated compressive stress expands to the fissure 2 right side, indicating that the tensile crack may initiate at tip B of fissure 1 and propagate to the fissure 2 right side. Moreover, there is a strong tensile stress concentration behavior in the straight-line zone between tip B of fissure 1 and tip D of fissure 2 when *β*_2_ = 145° and 180°, meaning that tensile crack is most likely to form in the straight-line zone of BD.

Fig 11 (b) shows the parallel bond force distribution before the cracking of red sandstones with double unparallel prefabricated fissures based on DEM simulations [29]. Among them, the black line represents compressive stress and the red line represents tensile stress. The parallel bond force distribution with different *β*_2_ is completely consistent with that of major principal stress in Fig 11 (a), validating the stress distribution of the red sandstone with two unparallel prefabricated fissures before cracking under compression simulated by FDEM in this work.

**Fig 12:**
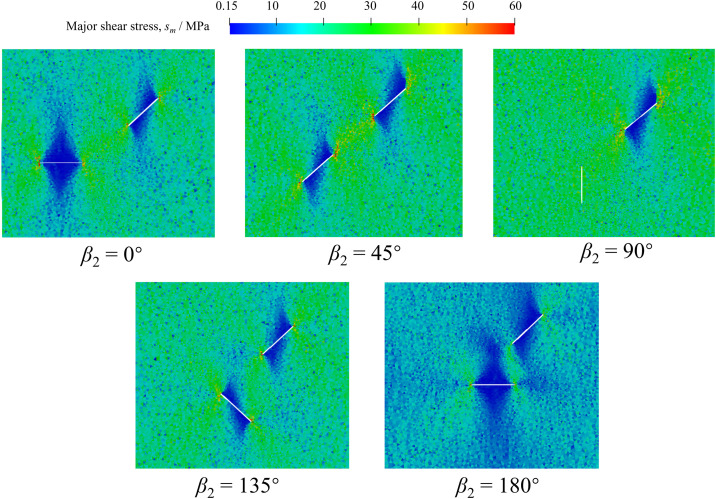
Distribution of major shear stress near the prefabricated fissures before cracking of red sandstones with different *β*_2_.

Fig 12 shows the distribution of major shear stress near the prefabricated fissures before cracking of red sandstones with different *β*_2_. A small major shear stress zone on both sides of the prefabricated fissures except for *β*_2_ = 90°. The extension direction of this zone is along with the loading direction, indicating that shear failure is impossible in this zone. At the same time, the major shear stress is concentrated at the tips of the two unparallel prefabricated fissures. Comparatively, the major shear stress at the fissure tips with *β*_2_ of 180° is much smaller than that of other cases, which characterizes that shear cracks are more difficult to form in this case. Particularly, shear cracks will not be formed around fissure 2 due to no shear stress concentration around fissure 2.

**Fig 13:**
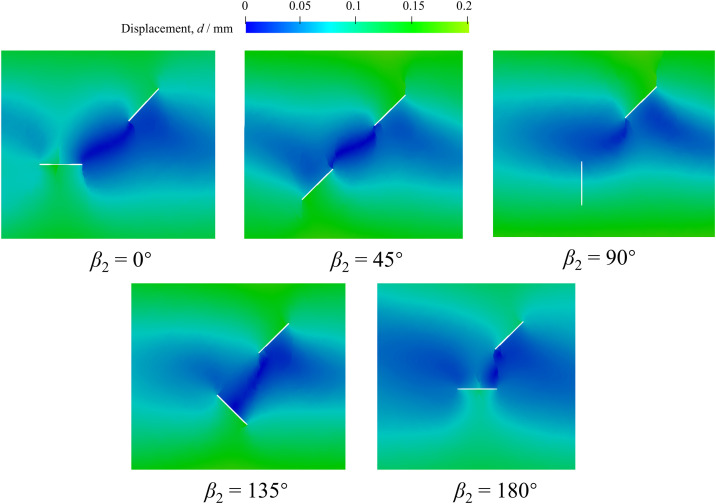
Displacement field near the prefabricated fissures before cracking of red sandstones with different *β*_2_.

Fig 13 presents the displacement field near the prefabricated fissures before cracking of red sandstones with different *β*_2_. The existence of prefabricated fissures significantly influences the displacement field of red sandstones under uniaxial compression. The displacement distribution becomes discontinuous because of prefabricated fissures. Since the equal loading rate is applied at the sample top and bottom, the smallest displacement of 0 appears at the sample middle. Furthermore, Fig 13 also shows that there is a minimum displacement field in the straight-line zone between the two unparallel prefabricated fissures in addition to *β*_2_ of 90°, while fissure 2 has little effect on the distribution of displacement field and only fissure 1 hinders the continuity of displacement field when *β*_2_= 90°.

**Fig 14:**
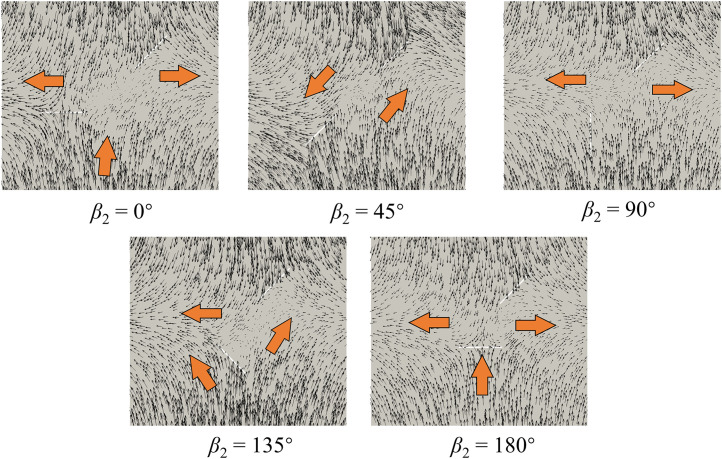
Displacement vector near the prefabricated fissures before cracking of red sandstones with different *β*_2_.

The displacement vector near the prefabricated fissures before cracking of red sandstones with different *β*_2_ is shown in Fig 14. Similarly, the orientation of fissure 2 has a great influence on the displacement vector near the prefabricated fissures. The specific characteristics are as follows. The fissure section changes the displacement vector direction from vertical to horizontal, especially for *β*_2_= 0° and 180°. In other words, the displacement vector direction is significantly changed when the fissure2 orientation is perpendicular to the loading direction. When *β*_2_= 45° and 135°, the influence of fissure 2 on the change of displacement vector is smaller than that with *β*_2_ of 0° and 180°. However, the existence of fissure 2 does not affect the displacement vector when *β*_2_= 90°, which presents the same distribution as that in other complete zones. Given the above analysis, the displacement vector distribution of red sandstone under uniaxial compression depends on the angle between the prefabricated fissure orientation and the loading direction.

### Failure mode

**Fig 15:**
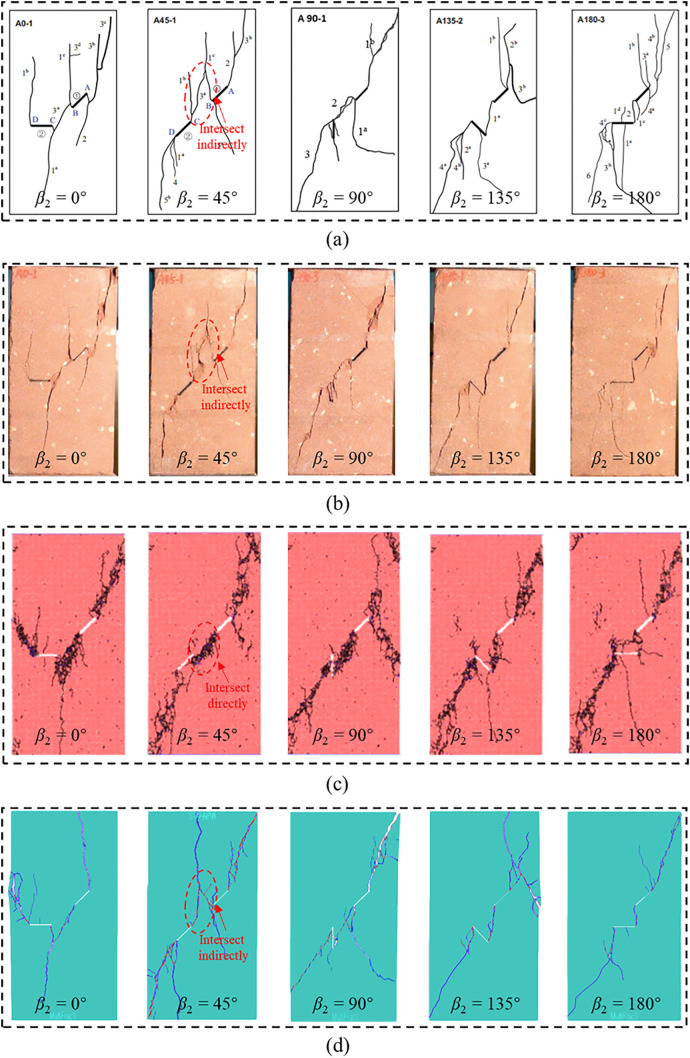
Comparison of the final failure modes obtained by (a) sketching, (b) experimental tests [9], (c) DEM [29], and (d) FDEM simulations.

Fig 15 presents the final failure modes of red sandstones with two unparallel prefabricated fissures under uniaxial compression. Fig 15 (a) is the sketched crack morphology based on the experimental results [9] (Fig 15 (b)). Meanwhile, Yang et al. [29] obtained the failure mode based on DEM modeling, as shown in Fig 15 (c). The failure modes obtained by FDEM in this paper is presented in Fig 15 (d). Overall, the final failure modes of red sandstones with prefabricated fissures under uniaxial compression obtained by different approaches are similar, and the orientation of fissure 2 greatly affects the final failure modes. However, FDEM can clearly reproduce the crack distribution characteristics observed in the test, but the crack morphology obtained by DEM is not very intuitive.

Combined with Figs 11 and 15, we find that the crack propagation and intersection are closely related to the distribution of major principal stress before cracking. Specifically, cracks often initiate and propagate in stress concentration zones. Therefore, the major principal stress distribution before material cracking can effectively predict the location of crack initiation and propagation and reveal the crack intersection mechanism. In addition, both the experimental and FDEM results show that cracks initiate and develop directly at the tips of the two unparallel prefabricated fissures during compression except for *β*_2_ = 45°. In the case of *β*_2_ = 45°, the tip B of fissure 1 and tip C of fissure 2 are not directly connected by new generated cracks, which develop at the top of the two fissures and then connect and intersect indirectly. This is because no stress concentration is observed in the straight-line zone between the two fissure tips when *β*_2_ = 45°, but the concentrated stress is mainly distributed at the top two fissures, as shown in Fig 11 (a). Nevertheless, DEM fails to simulate the indirect intersection behavior of cracks, which shows that cracks directly develop and intersect at the two prefabricated fissure tips when *β*_2_ = 45° (Fig 15 (c)). Consequently, the modeling results in this paper are more consistent with the experimental results than DEM results, which proves the effectiveness and superiority of FDEM in simulating rock cracking.

It is worth mentioning that rock damage caused by water-rock interaction is a hot topic in rock mechanics. Some previous studies indicated that the failure mode and crack propagation of fractured sandstone are determined by the high-humidity action time. The humidity action facilitates the evolution of resistance tensile cracks from wing cracks and keeps cracks from transforming to oblique shear cracks, which also causes the failure mode of fractured sandstone to move from shear to tensile damage [14,65]. However, the fracturing mechanism of sandstone containing unparallel prefabricated fissures under high humidity conditions is not clear. In the future, the FDEM humidity-fracture coupling model will be adopted to deeply study the mechanical behavior and failure mode of fractured sandstone in high humidity condition, and experimental results provided by Chen et al. [14,65] are expected to validate the FDEM modeling results.

### Energy evolution

**Fig 16:**
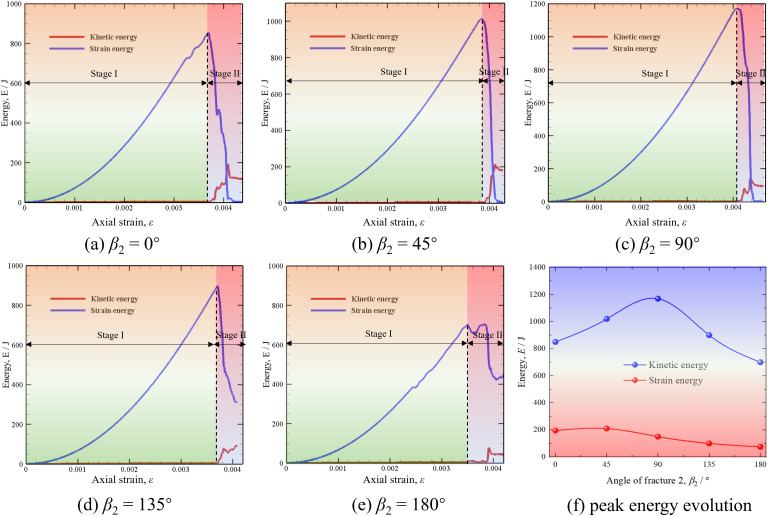
Energy evolution. (a)–(e) Evolution of strain and kinetic energy with axial strain in red sandstone with different *β*_2_, (f) relationship between the peak energy with *β*_2_.

Fig 16 illustrates the evolution of strain and kinetic energy with axial strain in red sandstone and the relationship between the peak energy with *β*_2_. In Fig 16 (a)–(e), the kinetic energy and strain energy with different *β*_2_ have the same evolution characteristics. Specifically, the strain energy has two evolution stages. The first stage is that the strain energy gradually accumulates at the beginning of the loading, which shows an increasing trend as the axial strain increases with a rising cumulative rate. The second stage is that the strain energy suddenly decreases to 0 after reaching the peak value, in which many cracks are generated, resulting in the release of most strain energy. Since the red sandstone studied in this paper is a brittle material, the strain energy is completely released in a very short time. The above description of the strain energy evolution applies to different *β*_2_, indicating that this feature depends largely on the material properties. The kinetic energy has three evolution stages. The first stage is that the kinetic energy is 0 because no damage occurs inside the sample. The second stage is the rapid rising of kinetic energy, which corresponds to the second evolution stage of strain energy. This is because many cracks initiate at this stage, and some strain energy is converted into kinetic energy. The kinetic energy reaches its peak value when the strain energy reduces to 0. The third stage of kinetic energy is a slight decrease to a stable development stage.

Fig 16 (f) illustrates the effect of *β*_2_ on the peak values of strain energy and kinetic energy. We can find that the peak strain energy increases first and then decreases with the increase of *β*_2_. The peak strain energy has the largest value with *β*_2_ of 90°, indicating that the system stores the most strain energy under external load. In this case, the sample has the highest UCS and Young’s modulus, as shown in Fig 8 (a) and (c). Compared with strain energy, the peak value of kinetic energy decreases with the increase of *β*_2_. In general, the peak value of strain energy is much higher than that of kinetic energy. This is because the loading displacement applied at each step is very small, and the system can be considered quasi-static during the loading process. Therefore, the kinetic energy generated by crack propagation and rock failure is much smaller than the strain energy stored in the sample under external load. This law is also consistent with the energy evolution characteristics of mudstone during water absorption using FDEM in our previous work [44].

The mechanical mechanism of above strain energy and kinetic energy evolutions can be explained as follows. The cracking process of fractured sandstone under uniaxial compression is essentially a dynamic evolution of energy among strain energy storage, transformation, and kinetic energy release. The increase in loading displacement directly drives the external force to do work, which is primarily converted into strain energy stored within the fractured sandstone. During the elastic stage, strain energy increases linearly with loading displacement, and the rock undergoes recoverable deformation [66]. As the loading displacement continues to increase, microcracks begin to nucleate and expand within the fractured sandstone, which requires energy consumption to overcome the rock’s cohesive force. At this point, the accumulation rate of strain energy begins to decrease, with a portion of the energy being used for crack formation and propagation [67]. When cracks reach critical length and enter the unstable propagation stage, a large amount of accumulated strain energy is instantaneously released. Part of this released energy will continue to be used for further crack propagation, but more importantly, it will manifest in the form of kinetic energy. This production of kinetic energy is due to the violent acceleration of the entire fractured sandstone when cracks suddenly propagate uncontrollably, such as through fragment ejection, shock wave propagation. Therefore, the increase in loading displacement is the driving force for energy input and strain energy accumulation, while crack propagation, especially unstable propagation, is the key link in the conversion of strain energy to kinetic energy [68]. When the crack propagation rate increases sharply, the kinetic energy also rises sharply, marking the destructive transition of the fractured sandstone from storing energy to releasing energy.

## Conclusions

In this paper, the mechanical behavior and fracturing mechanism of sandstone with two unparallel prefabricated fissures under uniaxial compression are studied using FDEM. After parameter calibration, a series of uniaxial compression numerical models of red sandstone samples containing two fissures with different angles (*β*_2_) are established in FDEM to study the influence of *β*_2_ on the stress-strain curve, crack number and acoustic emission (AE) characteristics, failure mode, stress and displacement field and energy evolution during uniaxial compression. The following conclusions can be drawn.

(1) FDEM can be effectively utilized to study the mechanical behavior and fracturing mechanism of red sandstone with two unparallel prefabricated fissures under uniaxial compression.
(2) The input parameters calibrated through the uniaxial compression test on intact red sandstone are validated by comparing the modeling stress-strain curves and failure modes with the experimental results.
(3) The uniaxial compressive strength, peak strain, and Young’s modulus all increase first and then decrease with the increase of *β*_2_. Especially, these indexes have a maximum value when *β*_2_ = 90°, and have the smallest value with a *β*_2_ of 180°. The sample exhibits progressive failure characteristics when *β*_2_ = 180° based on AE singles, while brittle failure is dominant in other cases. Generally, shear failure is concentrated at the crack tip, while tensile failure mainly occurs in the complete zone inside the specimen.
(4) The peak value of strain energy also shows a first increasing and decreasing trend with the increase of *β*_2_, but the kinetic energy decreases as *β*_2_ increases. A *β*_2_ of 90° can lead to the maximum strain energy peak.
(5) The simulated mechanical behavior, failure mode and crack morphology reproduce most of the phenomena observed in the laboratory test, demonstrating that the modeling results by FDEM are reasonable.

It should be pointed out that this paper only conducts a series of two-dimensional FDEM simulations and validates the simulation results with experimental results. However, the real rock samples are three-dimensional. In the future, we will perform three-dimensional FDEM numerical simulations on the mechanical behavior of fractured rock samples under uniaxial compression.

## Supporting information

S1 Table Statistical summary of UCS obtained by experiment and numerical simulation. (DOCX)

S2 Table Statistical summary of peak strain obtained by experiment and numerical simulation. (DOCX)

S3 Table Statistical summary of secant Young’s modulus obtained by experiment and numerical simulation. (DOCX)
