# Supplementary material for: Study on the mechanical behavior and fracturing mechanism of rock containing two unparallel prefabricated fissures under uniaxial loading
Source: PLoS One. 2026 Apr 17;21(4):e0347408. doi: 10.1371/journal.pone.0347408 (PMC13089698; doi:10.1371/journal.pone.0347408)
Supplement: S1 Table — (DOCX) [file pone.0347408.s001.docx]

**Statistical summary of UCS obtained by experiment and numerical simulation.**

| **Angle of fissure 2 (°)** | **Experiment 1 (MPa)** | **Experiment 2 (MPa)** | **Experiment 3 (MPa)** | **Numerical simulation (MPa)** |
| --- | --- | --- | --- | --- |
| 0 | 35 | 38 | 39 | 36 |
| 45 | 38 | 41.5 | 42 | 40 |
| 90 | 43 | 44.8 | 46 | 43.9 |
| 135 | 37 | 34.5 | 34 | 36.2 |
| 180 | 29 | 32 | 34.2 | 30.5 |
