# Supplementary material for: Study on the mechanical behavior and fracturing mechanism of rock containing two unparallel prefabricated fissures under uniaxial loading
Source: PLoS One. 2026 Apr 17;21(4):e0347408. doi: 10.1371/journal.pone.0347408 (PMC13089698; doi:10.1371/journal.pone.0347408)
Supplement: S2 Table — (DOCX) [file pone.0347408.s002.docx]

**Statistical summary of peak strain obtained by experiment and numerical simulation.**

| **Angle of fissure 2 (°)** | **Experiment 1** | **Experiment 2** | **Experiment 3** | **Numerical simulation** |
| --- | --- | --- | --- | --- |
| 0 | 0.00405 | 0.004 | 0.0038 | 0.004 |
| 45 | 0.0042 | 0.0044 | 0.0039 | 0.0042 |
| 90 | 0.0044 | 0.0043 | 0.00445 | 0.00442 |
| 135 | 0.0039 | 0.0042 | 0.0044 | 0.0041 |
| 180 | 0.0039 | 0.0042 | 0.0036 | 0.004 |
