# Supplementary material for: Study on the mechanical behavior and fracturing mechanism of rock containing two unparallel prefabricated fissures under uniaxial loading
Source: PLoS One. 2026 Apr 17;21(4):e0347408. doi: 10.1371/journal.pone.0347408 (PMC13089698; doi:10.1371/journal.pone.0347408)
Supplement: S3 Table — (DOCX) [file pone.0347408.s003.docx]

**Statistical summary of secant Young's modulus obtained by experiment and numerical simulation.**

| **Angle of fissure 2 (°)** | **Experiment 1 (GPa)** | **Experiment 2 (GPa)** | **Experiment 3**  **(GPa)** | **Numerical simulation** |
| --- | --- | --- | --- | --- |
| 0 | 9 | 9.5 | 9.8 | 9 |
| 45 | 8.4 | 9.7 | 11 | 9.5 |
| 90 | 10 | 9.9 | 10.5 | 10.2 |
| 135 | 9.2 | 8.7 | 8.2 | 9.1 |
| 180 | 7.8 | 8.5 | 8.8 | 7.6 |
